# Fermented *Cordyceps militaris* Extract Prevents Hepatosteatosis and Adipocyte Hypertrophy in High Fat Diet-Fed Mice

**DOI:** 10.3390/nu11051015

**Published:** 2019-05-06

**Authors:** Nguyen Khoi Song Tran, Goon-Tae Kim, Si-Hyun Park, Dongyup Lee, Soon-Mi Shim, Tae-Sik Park

**Affiliations:** 1 Department of Life Science, Gachon University, Sungnam, Gyeonggido 13120, Korea; marsnk217@gmail.com (N.K.S.T.); chng00@naver.com (G.-T.K.); 09050818@hanmail.net (S.-H.P.); nada_dongyup@naver.com (D.L.); 2 Department of Food Science and Biotechnology, Sejong University, Seoul 05006, Korea

**Keywords:** obesity, non-alcoholic steatohepatitis, fatty acid oxidation, fermentation, sphingosine 1-phosphate

## Abstract

Nonalcoholic fatty liver diseases (NAFLD) is characterized by accumulation of lipid droplets in the liver. The objective of this study was to evaluate protective effects of fermented *Cordyceps militaris* extract by *Pediococcus pentosaceus* ON188 (ONE) against hepatosteatosis and obesity in mice fed a high-fat diet (HFD). Eight-week-old male C57BL/6J mice were fed HFD mixed with ONE for four weeks and its effects on hepatosteatosis and obesity were examined. Although ONE did not change food intake, it reduced body weights of mice at administration dose of 200 mg/kg/day. Activities of lactate dehydrogenase (LDH), aspartate transaminase (AST), and alanine transaminase (ALT) as plasma parameters were reduced by ONE in a dose-dependent manner. Hepatic lipid droplets and triglyceride (TG) levels were also reduced by ONE due to upregulation of fatty acid oxidizing genes such as carnithine palmitoyltransferase (CPT1) and peroxisomal proliferator activated receptor α(PPARα) mediated by induction of sphingosine kinase 2 (SPHK2). In epididymal fat tissue, sizes of adipocytes were significantly reduced by ONE in a dose-dependent manner. This is mainly due to suppression of lipogenesis and upregulation of adipocyte browning genes. Collectively, these results suggest that fermented ONE can activate fatty acid oxidation via SPHK2 in the liver. It can also suppress lipogenesis and activate browning in adipose tissue. Thus, ONE might have potential to be used for the development of functional foods against liver dysfunction and obesity.

## 1. Introduction

Western diet and sedentary life-style are associated with development of chronic metabolic disorders including obesity, insulin resistance, and hepatosteatosis. Non-alcoholic fatty liver disease (NAFLD) encompasses a large spectrum of diseases ranging from simple steatosis (accumulation of hepatic triglyceride (TG)), nonalcoholic steatohepatitis (NASH), and fibrosis to cirrhosis [1,2]. Hepatosteatosis represents the very first stage with impaired fatty acid oxidation (FAO), abnormal fatty acid (FA) uptake via circulation, and increased de novo lipogenesis [3]. The following inflammatory stage, called non-alcoholic steatohepatitis (NASH), is characterized by hepatocyte injury, inflammatory infiltrate, and collagen deposition [4,5]. Afterward, cirrhosis and hepatocellular carcinoma are developed [6]. To develop therapeutic methods against hepatosteatosis, many studies have been conducting targeted TG reduction by activating FAO in liver and browning of white adipose tissue (WAT) for heat production [3,7].

FAO is a process that shortens fatty acids into acetyl-CoA which can be later converted to ketone bodies or fully oxidized in the TCA cycle [8]. Peroxisome proliferator-activated receptor (PPAR) α and its co-activator PPARγ co-activator 1 (PGC-1) α are critical in enhancing expression of target genes including carnitine palmitoyl transferase 1 (CPT1) and acyl-CoA oxidase (ACOX) [9,10]. Adenoviral expression of sphingosine kinase 2 (SPHK2), a major isoform in the liver, can activate FAO and improve hepatosteatotic condition in diet-induced obese mice model [5]. Brown adipose tissue (BAT), an organ of thermogenesis, possesses uncoupling protein 1 (UCP1) in the inner membrane of its mitochondria that can generate heat from mitochondrial oxidation, thus causing energy expenditure and conferring metabolic benefit. BAT has drawn attention for its therapeutic role in obesity and metabolic disorders. Recent discoveries have demonstrated that UCP1 can be a target for WAT browning in response to physiological or diet-induced stimuli [11,12,13]. Various regulators including sirtuin 1(SIRT1)-mediated deacetylation of PPARγ and PR domain zinc finger protein 16 (PRDM16) have been discovered to be able to stimulate browning of WAT [14,15].

*Cordyceps militaris* (CM), a well-known traditional medicinal mushroom, has been applied as functional food in East Asia [16]. CM has been suggested as an efficacious medicine for eternal youth for its protective effects on mitochondria, testosterone stimulation, and aging [17,18]. CM is known to possess anti-oxidant, anti-inflammatory, and anti-cancer activities [19,20,21,22]. Among the components of CM, adenosine and cordycepin have been found to play important roles in modulating hepatosteatosis and atherosclerosis [23,24,25]. However, mechanisms involved in the effect of CM supplementation on hepatosteatosis have not been fully elucidated since its preventive effect is not profound.

In this study, we investigated effects of *Pediococcus pentosaceus*-fermented CM on FAO in liver and browning of WAT of mice fed a high fat diet (HFD). Surprisingly, we found the involvement of sphingolipid metabolism as well as inducible BAT in prevention of hepatosteatosis process.

## 2. Materials and Methods

### 2.1. Preparation of ONE and Chow

As described in a previous study [2], an authenticated voucher specimen of *Cordyceps militaris* was obtained from the Herbarium at the College of Bioscience and Biotechnology, Konkuk University (Seoul, Korea). After inoculating on germinated soybeans (*Rhynchosia volubilis Lour*), CM was cultured at 20–25 °C for 4 weeks (germinated soybean-grown cordyceps, GSC). Harvested GSC were dried and ground to powder and extracted with boiling water for 2 h. For fermentation, *Pediococcus petosaceus* ON188 was inoculated in CM water extract and incubated at 37 °C for 48 h. Supernatants of extracts were filtered and dried with a rotary evaporator under vacuum at 40 °C and freeze-dried. The powder was stored at −20 °C and mixed with mouse chow from Dooyeol Biotech (Seoul, Korea). There were three doses of treatment: ONE50, ONE100, and ONE200 representing doses of 50, 100, and 200 mg of *P. pentosaceus* ON188-fermented GSC extract (ONE)/kg/day, respectively.

### 2.2. Gass Chromatography-Time of Flight Mass Spectrometry (GC-TOF MS) Analysis

Adenosine and cordycepin were quantified using the method described previously [26]. Briefly, whole GSC extract fermented with *P. pentosaceus* ON188 (ONE) were derivatized by N-methyl-N-trimethylsilyltrifluoroacetamide (MSTFA with 1% TMCS, Thermo) for trimethylsilylation [27]. A 0.5μL of derivatized mixture was injected using an Agilent 7693 ALS (Agilent Technologies, Wilmington, DE, USA) in splitless mode into an Agilent 7890B gas chromatograph (Agilent Technologies, Wilmington, DE, USA) for chromatographic separation using Rtx-5Sil MS column. Mass spectrometric analysis was conducted on a LECO Pegasus HT time-of-flight (TOF) mass spectrometer controlled by LECO ChromaTOF software version 4.50 (LECO, St. Joseph, MI, USA). Mass spectra were collected from 85 to 500 m/z at acquisition rate of 17 spectra/second of and detector voltage of 1800 V. Data pre-processing was conducted using ChromaTOF software upon data acquisition in which apex mass values, the entire spectrum, retention time, peak purity, and signal-to-noise ratio were acquired [28].

### 2.3. Animal Experiments

Six-week-old male C57BL/6J mice were obtained from Raon bio (Gyeonggi-do, Korea). All mice were maintained in a specific pathogen-free facility with 12:12 h light/dark cycle. Water and normal chow diet (NCD) were given ad libitum for 1 week. After adjustment period, mice were fed a high fat diet for 1 week and then divided into 6 groups (*n* = 7): (1) Control groups (NCD); (2) 60% kcal high-fat diet (HFD); (3) 20 mg/kg/day fenofibrate; and treatment groups of (4) ONE50, (5) ONE100, and 6) ONE200 representing 50, 100, and 200 mg of ONE/kg/day (mpk) for 4 weeks, respectively. Body weights of mice were checked every week before sacrifice. All experimental procedures were approved by Gachon University Institutional Animal Care and Use Committee (IACUC).

### 2.4. RNA Preparation and Real Time Quantitative PCR

Total RNAs were extracted from liver and adipose tissues using mRNA Extraction Kit (Intron Biotechnology Inc., SeungNam, Korea) according to the manufacturer’s procedure. Complementary DNA was synthesized using iScript cDNA synthesis Kit (Bio-Rad, Hercules, CA, USA) in a PCR Thermal Cycler (TaKaRa, Japan). Quantitative real-time PCR (qRT-PCR) analysis was performed using SYBR Green Premix (TaKaRa, Japan) in Step-One Plus real-time PCR system (Applied Biosystems, Carlsbad, CA, USA). Subsequently, mRNA level was normalized to mouse GAPDH and determined by the 2^−ΔΔCt^ method. Primer sequences used in this study are listed in Table S1.

### 2.5. Protein Assay and Western Blot Analysis

After four weeks of feeding, six groups of mice (NCD, HFD, Fenofibrate, ON50, ON100, and ON200) were sacrificed to collect liver and adipose tissues. Murine liver and fat tissues were homogenized in 1 mL of RIPA buffer (10 mM Tris pH 7.8, 1 mM EDTA, 150 mM NaCl, phosphatase inhibitor, and protease inhibitor) using a homogenizer. Tissue lysate was collected and protein analysis was performed using Protein Assay Kit (Bio-Rad, Hercules, CA, USA). Total protein (40 µg) was then subjected to sodium dodecyl sulfate polyacrylamide gel electrophoresis (SDS-PAGE) and PVDF membrane transfer (Millipore, Bedford, MA, USA). Rabbit primary antibodies against carnitine palmitoyltransferase (CPT1α), UCP1α, adipocyte protein 2 (AP2), PPARγ, CCAAT/enhancer-binding protein α (c/EBPα) (Cell Signaling Technology, MA, USA), PPARα, peroxisomal acyl-coenzyme oxidase 1 (ACOX1), and SPHK2 (Abcam, Cambridge, MA, USA) were then used respectively to detect target proteins on PVDF membrane. These samples were normalized to mouse primary antibody against β-actin (Millipore, Cambridge, MA, USA). After incubation with secondary horseradish peroxidase (HRP) conjugated goat anti-rabbit IgG antibody (Millipore, Bilerica, MA, USA), blots were developed with enhanced chemiluminescent substrate (Millipore, Bilerica, MA, USA). Bands were detected using a Chemiluminescence imaging equipment (Viber Lourmat, France).

### 2.6. Plasma and Liver TG Level

After four week of HFD and 16 h of fasting, blood glucose level at basal condition was measured using an automatic glucose monitor (One Touch, Lifescan, Milpitas, CA, USA). Plasma and hepatic TG, cholesterol, HDL, and LDL levels were determined using colorimetric assay kits from Abcam (Cambridge, MA, USA) while lactate dehydrogenase (LDH), alanine transaminase (ALT), and aspartate transaminase (AST) levels were measured using an Activity assay kit (Sigma Aldrich, St. Louis, MO, USA) as described previously [5].

### 2.7. Sphingolipids Analysis

Approximately 100 mg of liver was used for analyses of sphingolipids. A known amount of C17:0–ceramide was added as an internal standard to tissue extracts containing 1 mg of proteins. Sphingolipids were extracted using chloroform/methanol (2:1, v/v) containing 0.01% butylated hydroxytoluene. KOH was added to the mixture in order to saponify glycerolipids at 37 °C for 2 h. Lipid extracts were neutralized by adding acetic acid. The organic phase was separated and dried under nitrogen gas. Sphingosine 1-phosphate (S1P) was separated by HPLC with a C18 column (XTerra C18, 3.5 um, 2.1 × 50 mm) and ionized in positive electrospray ionization mode as previously described by Yoo et al. [29] with modifications. [M+]/product ions from corresponding to sphingolipid metabolites were subjected to multiple reaction monitoring (MRM) quantification using a bench-top tandem mass spectrometer, API 4000 Q-trap (Applied Biosystem, Framingham, MA, USA) interfaced with an electrospray ionization source.

### 2.8. Tissue Histology

Murine liver and white adipose tissues were collected and fixed in 4% formaldehyde for 24 h. Tissue sections (5 μm in thickness) were paraffin embedded and stained with hematoxylin and eosin (H&E, Sigma Aldrich). Images of stained sections were captured using a slide scanner [30].

### 2.9. Statistical Analyses

Results are expressed as mean ± SEM (standard error to the mean). Statistical significance between controls and treated groups was assessed using one-way analysis of variance (ANOVA) with Bonferroni’s multiple comparison post-hoc analysis using Prizm (GraphPad software, San Diego, CA, USA). *p*-values lower than 0.05 were considered significant.

## 3. Results

### 3.1. ONE Contains Adenosine and Cordycepin

**Figure 1:**
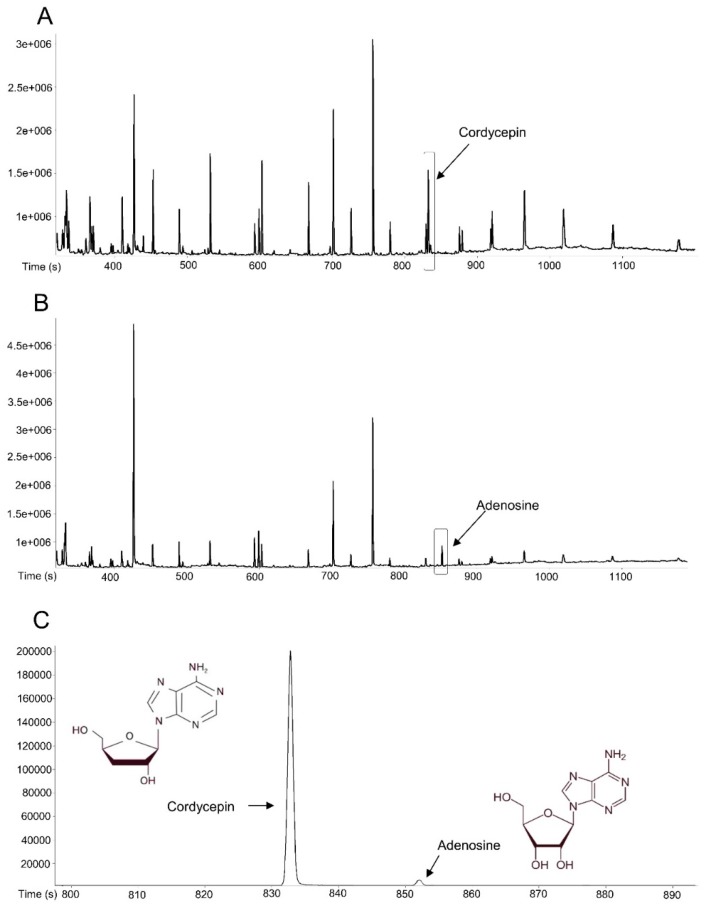
Ion chromatograms of adenosine and cordycepin. GC/MS analysis results of adenosine and cordycepin in ONE (236 m/z) are presented in total ion chromatograms of (**A**) cordycepin and (**B**) adenosine compared to (**C**) extracted ion chromatogram.

Previous reports have suggested that adenosine and cordycepin as major bioactive components in CM possess anti-steatotic effects [23,31]. Therefore, we examined contents of these two compounds in ONE using GC-TOF MS (Figure 1). Total ion chromatograms of cordycepin and adenosine standard are presented in Figure 1A,B. Extracted ion chromatogram of ONE is shown in Figure 1C. Major peaks of cordycepin and adenosine of standards in the ion chromatogram (positive ion mode 236 m/z) were eluted at retention time of 832.6 s and 851.75 s, respectively. Levels of adenosine and cordycepin in ONE were at 68.9 ± 0.57 μg/g and 1.36 ± 0.01 μg/g, respectively.

### 3.2. ONE Diet Decreases Body Weight Increase of Mice Fed HFD with no Change In Their Food Intake

**Figure 2:**
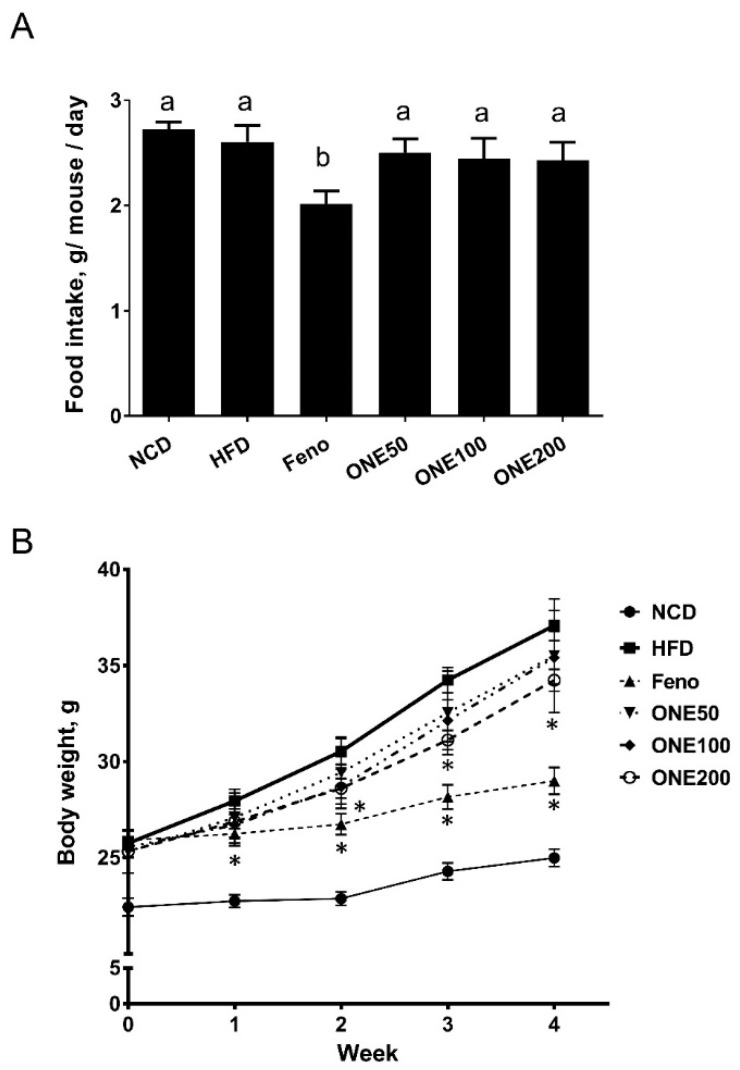
Food intake and body weight of mice fed with high fat diet (HFD) and ON188 (ONE). After one week of adjustment, mice were divided into six groups: Normal chow (NCD), high fat diet (HFD), fenofibrate (Feno), ONE50 (ONE at 50 mg/kg/day), ONE100 (ONE at 100 mg/kg/day), and ONE200 (ONE at 200 mg/kg/day). (**A**) Average food intake and (**B**) body weight were measured during 4-week of Feno and ONE co-feeding period. All values are presented as mean ± SEM (*n* = 7), * *p* < 0.05 vs. HFD feeding group. Different letters indicate statistical significance among treatment groups.

To examine whether ONE could prevent body weight increases in mice fed HFD, various doses of ONE were administered to mice via diet-admix (50, 100, 200 mpk). As a positive control, fenofibrate as a well-known peroxisomal proliferator activating receptor (PPAR) α agonist for FAO activation was administered to mice fed HFD (Feno, 20 mpk). Mice treated with ONE had similar appetite to those fed with NCD- and HFD-diet as control. Only mice treated with fenofibrate had 25% less food intake than those in the control HFD group (Figure 2A).

Mice fed HFD increased their body weight in a time-dependent manner. These increases were faster than those in the NCD group. In contrast, mice treated with fenofibrate showed slower body weight increases mainly due to decreased food intake. Mice treated with ONE at 200 mpk showed lower body weights from two weeks after treatment compared to those in the HFD group (Figure 2B). These results suggest that ONE has no effect on food intake, although it can decrease body weight increase at high dose.

### 3.3. ONE Prevents Systemic Tissue Damage and Improves Liver Function

Plasma biochemical parameters of six different treatment groups of mice were analyzed. Results are shown in Table 1. While the fenofibrate group showed reduced plasma glucose levels compared to the HFD group, there was no significant difference in blood glucose level between HFD control and ONE-treated groups. Similarly, the fenofibrate group showed decreased plasma cholesterol levels compared to the HFD group, but there was no significant difference in plasma cholesterol level between HFD and ONE-treated groups. When ONE was used at higher doses (100 and 200 mg/kg/day), elevated triglyceride (TG) levels were found, although LDL and HDL levels showed no significant changes.

Lactate dehydrogenase (LDH) activity was dramatically decreased by treatment with ONE in a dose-dependent manner, suggesting that overall tissue damage was protected by ONE [32,33]. Consistently, enzyme activities of aspartate transaminase (AST) and alanine transaminase (ALT) were drastically dropped in plasma samples of mice treated with ONE. These results suggest that overall tissue damage can be prevented and protected by ONE treatment, especially in the liver.

### 3.4. ONE Reduces Lipid Droplets in Livers of Mice Fed HFD

**Figure 3:**
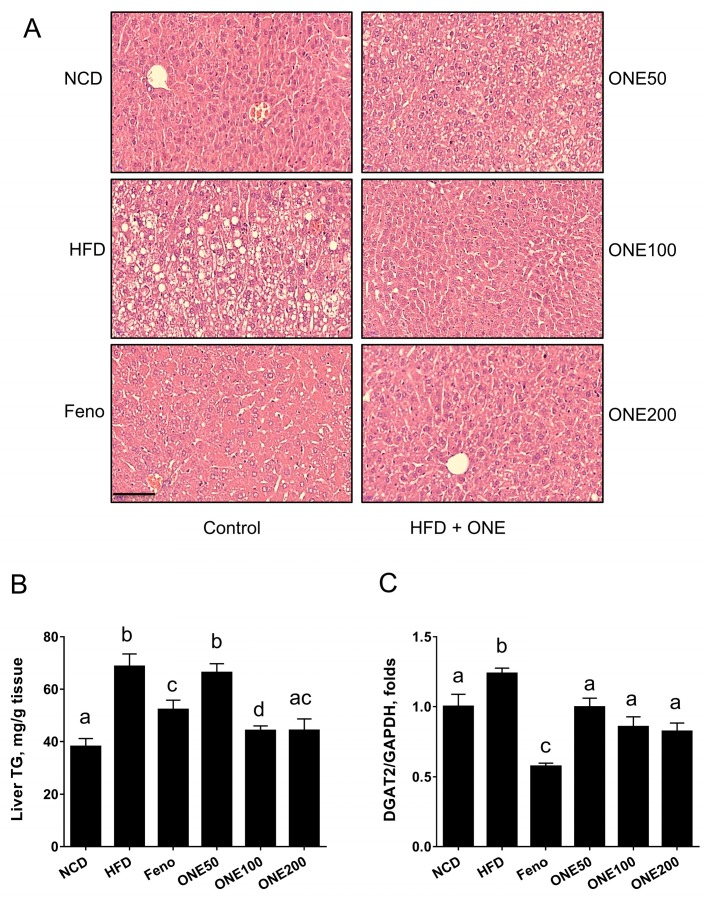
ONE prevents hepatosteatosis in mice fed HFD. After adjustment for one week, mice fed NCD or HFD were treated with fenofibrate or various doses of ONE. After sacrifice, mouse livers were stained with hematoxylin and eosin (H&E) (**A**). TG levels in livers were measured using colorimetric enzymatic method (**B**). Diglyceride acyltransferase (DGAT2) mRNA expression was determined using qRT-PCR (**C**). All values are presented as mean ± SEM (*n* = 7). Different letters indicate statistical significance among treatment groups. Bar indicates 100 μm.

Livers from each treatment group were isolated and their sections were stained with H&E to examine whether the occurrence of lipid droplets was altered by ONE. We found that HFD feeding increased lipid droplets and pathological states that resembled the occurrence of hepatoseatosis, including accumulation of lipid droplets and ballooning (Figure 3A). In contrast, livers isolated from mice fed NCD or fenofibrate showed no steatosis. When mice were treated with ONE at 50 mg/kg/day, ballooning and small lipid droplets were found in livers. However, they were significantly reduced compared to those in the HFD group. After treatment with ONE at doses of 100 and 200 mg/kg/day, these lipid droplets and ballooning disappeared.

Next, we quantitated the hepatic TG levels biochemically to examine whether pathophysiological condition of liver is associated with hepatic TG levels. The fenofibrate group has reduced hepatic TG levels compared with those of HFD group (Figure 3B). While TG levels in the livers of ONE50 group was not different from those in HFD group, TG levels in the livers of ONE100 or ONE200 group was reduced to the levels found in NCD group (Figure 3B). These results suggest that ONE treatment can decrease hepatic TG levels and improve hepatosteatotic conditions caused by HFD.

### 3.5. ONE Upregulates FA Oxidation Genes and SPHK2 in Liver

To explain how ONE altered hepatic TG, we first examined expression levels of genes in FA/TG biosynthetic pathway. We found that HFD feeding slightly upregulated diglyceride acyltransferase (DGAT2), a TG synthesizing enzyme from diacylglycerol. In contrast, the fenofibrate group showed downregulated DGAT level than the NCD group. ONE treatment also downregulated DGAT expression to levels in the NCD group (Figure 3C). However, the expression levels of other genes in the FA/TG biosynthetic pathway such as steatoyl CoA desaturase (SCD1) or fatty acid synthase were not significantly altered by treatment with ONE (data not shown).

**Figure 4:**
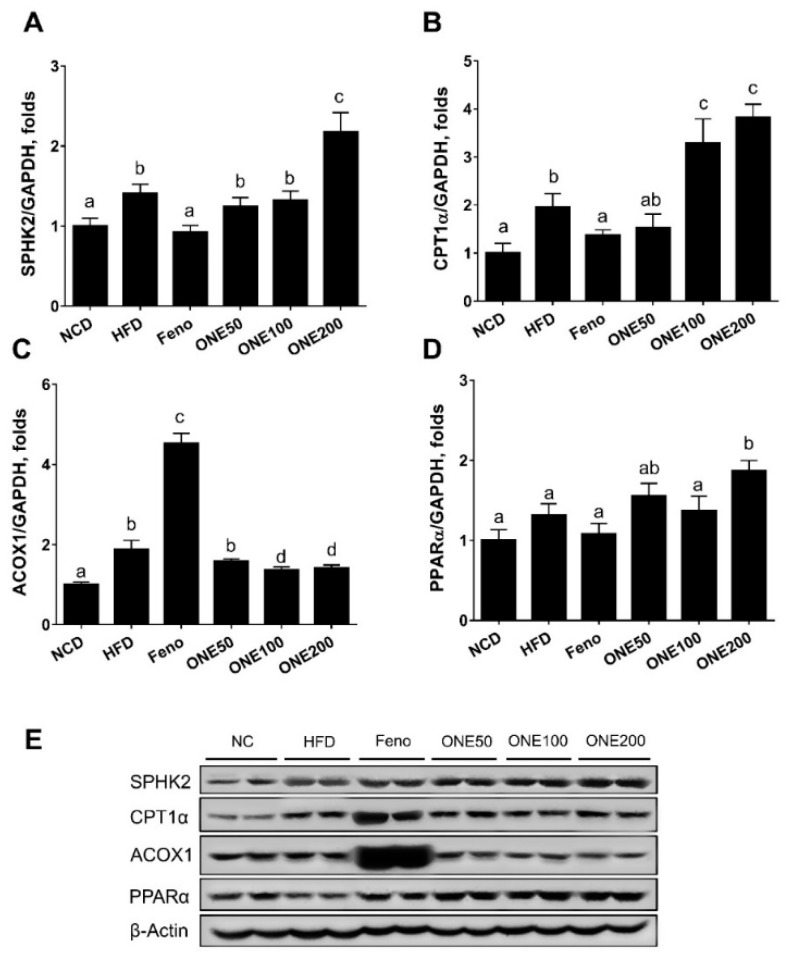
Upregulation of fatty acid oxidizing genes via sphingosine kinase 2 (SPHK2) activation in liver. After four weeks of co-feeding with ONE, mouse livers from six different treatment groups were collected and mRNA expression levels of (**A**) SPHK2, (**B**) carnitine palmitoyltransferase (CPT1α), (**C**) peroxisomal proliferator activated receptor α (PPARα), and (**D**) peroxisomal acyl-coenzyme oxidase 1 (ACOX1) were measured using qRT-PCR. Data are presented as mean ± SEM (*n* = 7). Different letters indicate statistical significance among treatment groups. (**E**) Protein levels of SPHK2, CPT1α, ACOX1, and PPARα were determined by immunoblot analyses method.

We have previously demonstrated that adenoviral overexpression of hepatic sphingosine kinase 2 (SPHK2) can activate FA oxidation in mice [5]. To examine whether SPHK2 might be associated with decreased hepatic lipid droplets, we measured expression levels of SPHK2 and FA oxidizing genes such as carnitine palmitoyltransferase (CPT1α), peroxisomal acyl-coenzyme oxidase 1 (ACOX1), and PPARα in the liver. Both mRNA and protein levels of SPHK2 were upregulated in livers of mice treated with diet containing 200 mpk ONE (Figure 4A,E) compared to those in the livers of mice fed HFD. However, fenofibrate suppressed the expression of SPHK2 to levels similar to those in the NCD group. CPT1α was upregulated by ONE treatment. However, no significant change was found in ACOX1 level after ONE treatment (Figure 4B,C). PPARα was only upregulated by ONE at 200 mpk (Figure 4D). Although we observed a two-fold increase in CPT1α mRNA expression level in livers of mice fed ONE200, there was no significant change in protein expression of CPT1α or ACOX1. However, PPARα was upregulated by ONE treatment compared to that in the HFD group. In contrast, fenofibrate group showed increased protein levels of CPT1α and ACOX1 compared to those in the HFD group (Figure 4E). These results suggest that upregulation of genes in FA oxidation pathway by ONE might have contributed to the reduction of lipid droplets in mouse liver.

### 3.6. ONE Reduces Cell Sizes of Adipocytes in Visceral White Adipose Tissues

**Figure 5:**
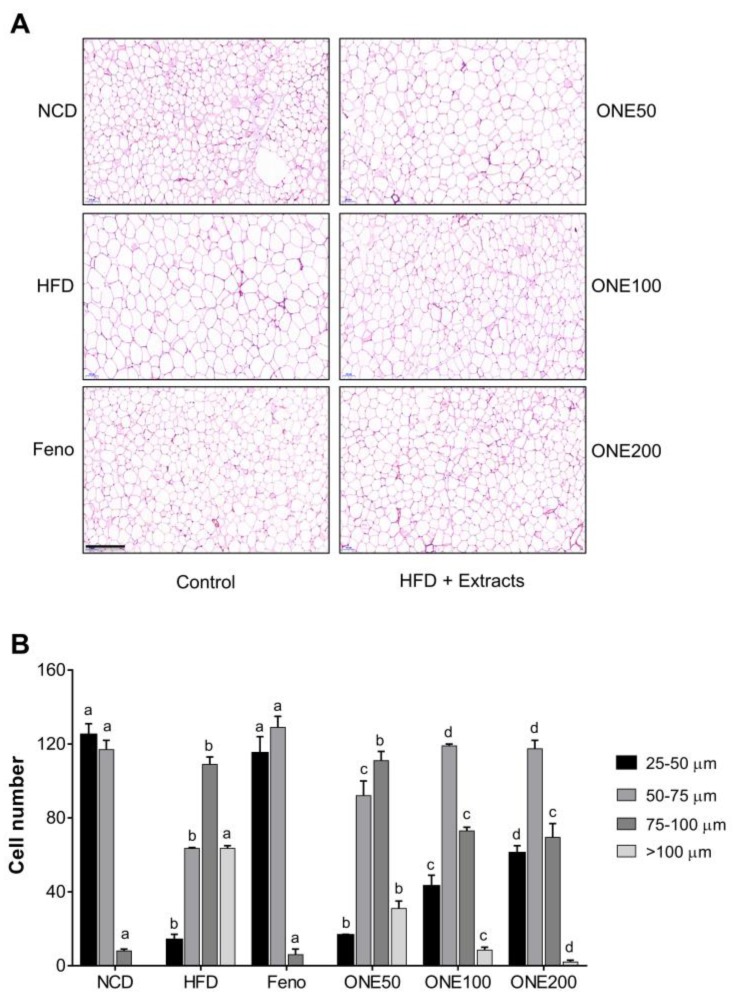
ONE diet reduces the size of adipocyte in white adipose tissues (WAT). (**A**) After four weeks of co-feeding with ONE, mouse WATs were collected and stained with H&E. (**B**) Number of adipocytes with various sizes (25–50, 50–75, 75–100, >100 μm) were counted. Data are presented as mean ± SEM (*n* = 7). Different letters indicate statistical significance among treatment groups. Bar indicates 100 μm.

To examine the effect of ONE on adipogenesis in vivo, we isolated visceral white adipose tissues (WAT) from mice and stained them with H&E. Adipocytes in the HFD group were hypertrophic and bigger than those in the NCD group due to lipid accumulation (Figure 5). When mice were treated with fenofibrate, the sizes of adipocytes were decreased, but not to levels as low as those in the NCD group. When mice were treated with ONE, sizes of adipocytes were reduced in a dose-dependent manner. To examine this in a quantitative manner, the number of cells at certain range of sizes was counted. While most adipocytes had a diameter less than 75 μm in NCD and fenofibrate groups, the majority of cells in the HFD group had a diameter of more than 75 μm (Figure 5B). Clear transition from cell size bigger than 75 μm to cell size smaller than 75 μm was observed in the group treated with ONE in a dose-dependent manner. These results suggest that ONE can prevent lipid accumulation and hypertrophy of adipocytes.

### 3.7. ONE Suppresses Expression of Genes Involved in Lipogenesis and Adipocyte Differentiation

**Figure 6:**
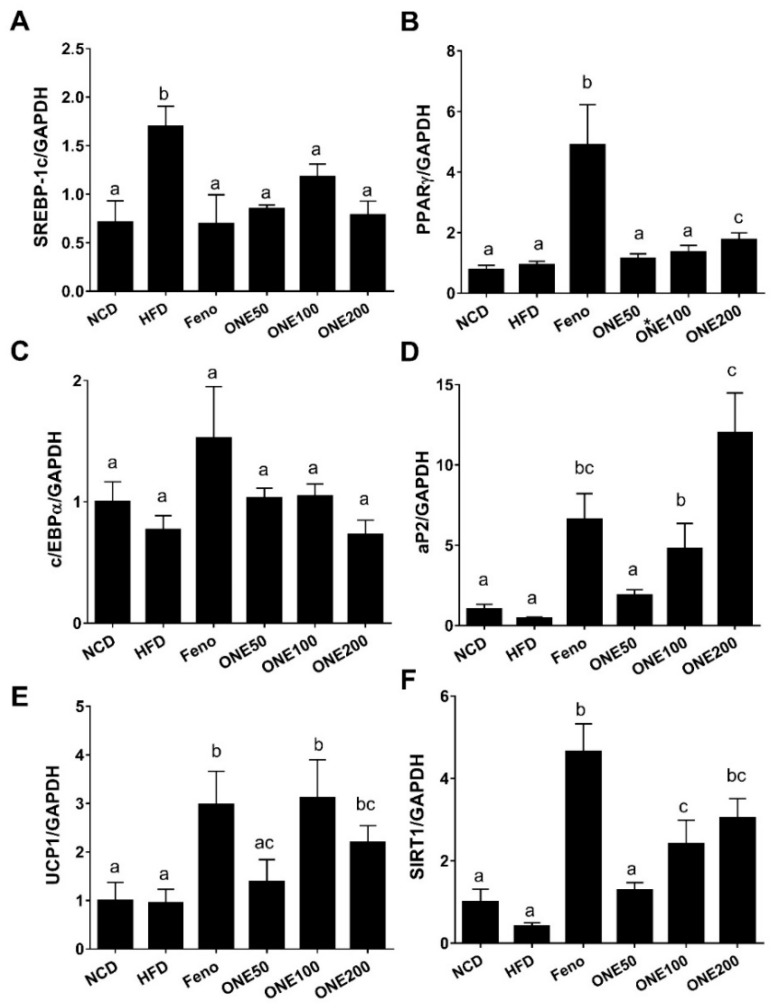
ONE suppresses expression of lipogenic and adipogenic genes but upregulates the expression of browning genes. After four weeks of ONE administration, mouse WATs were collected. Expression levels of lipogenic gene ((**A**) SREBP-1c), adipogenic genes ((**B**) PPARγ, (**C**) CCAAT/enhancer-binding protein α (c/EBPα) and (**D**) aP2), and browning genes ((**E**) uncoupling protein 1 (UCP1), (**F**) sirtuin 1 (SIRT1)) were measured using qRT-PCR. Results are presented as mean ± SEM (*n* = 7). Different letters indicate statistical significance among treatment groups.

Since we found that hypertrophy of adipocytes was inhibited by treatment with ONE in mice fed HFD, we examined whether ONE could regulate lipogenesis and adipogenesis in adipose tissues. First, we measured transcriptional levels of sterol response element binding protein-1c (SREBP-1c), a regulatory lipogenic protein for FA/TG biosynthesis. Although HFD upregulated SREBP-1c, treatment with ONE or fenofibrate suppressed its expression levels to reach levels in NCD group (Figure 6A). We then measured expression levels of adipogenic genes such as PPARγ, CCAAT/enhancer-binding protein α (c/EBPα), and adipocyte protein 2 (aP2). We found that expression level of PPARγ or c/EBPα was not significantly altered by treatment with ONE except for PPARγ (1.8-fold) in the ONE200 group. Only fenofibrate upregulated PPARγ by 4.9-fold (Figure 6B,C). Adipose protein 2 (aP2), another adipogenic gene, was upregulated by ONE to levels similar to those in the group treated with fenofibrate (Figure 6D). Since uncoupling protein 1 (UCP1) is a major protein that can reduce fat mass in brown and beige adipose tissue (BAT), we measured the expression level of UCP1. We found that ONE upregulated UCP1 to levels similar to those in the fenofibrate group (Figure 6D). SIRT1, another major gene important in BAT, was upregulated by fenofibrate and ONE (Figure 6E,F).

**Figure 7:**
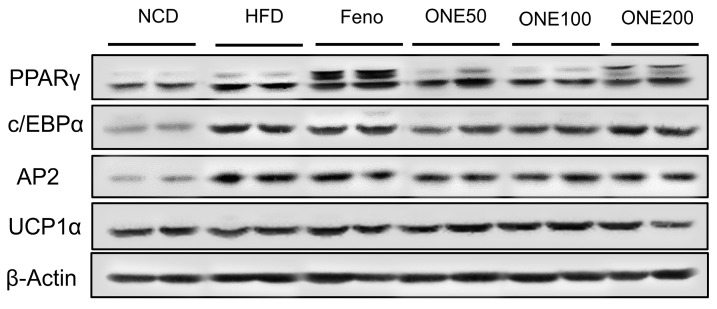
Protein expression levels of PPARγ, c/EBPα, aP2, and UCP1α were determined by immunoblot analyses method after four weeks of treatment with NCD, HFD, fenofibrate, or various concentrations of ONE.

To examine whether translational expression was consistent with mRNA expression, we measured protein levels by immunoblotting analyses. While adipose tissues isolated from mice treated with fenofibrate showed drastic increases of PPARγ protein level, no significant change in protein level of PPARγ was found in NCD, HFD, or ONE group (Figure 7). In contrast, c/EBPα and aP2 protein levels were reduced in ONE-treated group at all doses. Different from mRNA expression results, we did not find any difference in UCP1 protein expression. These results indicate that the reduction in size of adipocyte is mainly due to suppression of lipogenic and adipogenic genes by ONE treatment.

## 4. Discussion

Obesity is the major cause of chronic diseases including insulin resistance, cardiovascular disease, and NAFLD. When excess FA from WAT is spilled into bloodstream, TG is accumulated in non-adipose organs such as liver, skeletal muscle, and heart, causing steatosis [34]. In a state when FA uptake and synthesis exceed FA oxidation and secretion, lipid droplets are formed and accumulated in the liver. Although this early step of NAFLD has no clinical symptoms yet, it can lead to the development of insulin resistance, dyslipidemia, cardiometabolic risk, and certain NAFLD associated factors [35]. Steatosis is known to cause oxidative stress, mitochondrial dysfunction, production of pro-inflammatory cytokines, and apoptosis. It can lead to an advanced stage called nonalcoholic steatohepatitis (NASH) that can later progress to cirrhosis and hepatocellular carcinoma [36,37]. Our in vivo results suggest that: (1) ONE can protect mice with HFD against early stage of hepatosteatosis and obesity, (2) ONE can activate FAO in liver via upregulation of SPHK2, and (3) ONE induces expression of trans-differentiated and browning genes in WAT.

A traditional medicine in East Asia, *Cordyceps militaris* (CM) is well-known for its anti-inflammation, anti-anemia, and anti-cancer effects [16]. Recently, CM water extract has been shown to be able to decrease hepatic TG and total lipid contents in ob/ob mice [38]. Additionally, CM comprises of numerous components with anti-inflammatory and immune-modulating activities [39]. Adenosine and cordycepin in CM extracts have been suggested as phenotypic switches of macrophages in inflammatory response [40]. Although specific functions of adenosine in hepatosteatosis is controversial, recent reports have demonstrated that cordycepin and adenosine receptor 2A play important roles in hepatotoxicity and obesity-associated NAFLD [41,42,43]. Our recent study reveals that CM and GSC extracts possess high concentration of cordycepin and adenosine, but they do not affect expression of FAO genes [44]. We developed a method to culture CM in germinated soybeans (*Rhynchosia volubilis Lour*) (GSC) and fermented extracts using *Pediococcus pentosaus* ON188. It has been shown that GSC has high protective activities against cell cycle arrest and inflammatory response [2,45] while *Pediococcus* spp. can benefit human digestive and immune systems [46]. Thus, ONE was examined as a food therapy for hepatosteatosis.

Herein, we found that hepatosteatosis developed in mice fed HFD could be prevented by ONE treatment. Although treatment with ONE did not alter food intake or body weight, it significantly decreased sizes of adipocytes in a dose-dependent manner. This result suggests that TG accumulation in adipocytes could be inhibited by ONE. In contrast, fenofibrate treatment reduced body weight mainly by decreasing food intake. It is difficult to determine whether this inhibition of adipocyte hypertrophy could be reflected in prevention of adiposity or weight reduction at this moment because we only fed mice with HFD for four weeks. Long-term treatment of ONE should be studied further to verify the anti-obesity efficacy of ONE.

When plasma parameters were measured, ONE did not alter blood glucose, cholesterol, LDL, or HDL level in the liver compared to those in the HFD group. In contrast, enzyme activities of LDH, AST, and ALT were significantly reduced in ONE treated group. Especially, the highest ONE dose group clearly showed reduced values. These results are mainly due to protective effects of ONE against tissue damage, especially hepatic dysfunction. Preservation of liver function in mice fed HFD and ONE could be due to drastic removal of lipid droplets in the liver. At lower doses of ONE, hepatocytes only showed whitening and ballooning while most of lipid droplets disappeared. In consistent with this result, whitening or ballooning of hepatocytes was not found and morphology was more similar to that in the NCD group when mice were treated with ONE at higher dose. This is mainly due to transcriptional upregulation of CPT1α and PPARα and activation of FA oxidation. Interestingly, we did not find increased protein levels of FA oxidizing genes except for PPARα. We have previously demonstrated that adenoviral overexpression of SPHK2 in liver can activate hepatic FA oxidation and reduce lipid droplets in livers of mice fed HFD [5]. Upregulation of SPHK2 by ONE could be a major mechanism for the reduction of hepatic lipid droplets. SPHK2 upregulation-mediated activation of FA oxidation in liver could be a new therapeutic strategy to reduce hepatosteatosis.

HFD caused hypertrophy of adipocytes due to intracellular accumulation of lipid droplets. We found that ONE decreased cell sizes in a dose-dependent manner. Cell sizes after ONE treatment were comparable to those of the fenofibrate group. This is partly due to suppressed SREBP1c-mediated inhibition of de novo lipogenesis by ONE in visceral WAT. In contrast, adipogenesis mediated by PPARγ or c/EBPα was not affected. Rather, aP2 was upregulated by ONE, similarly to that upregulated by fenofibrate. Therefore, inhibition of adipogenesis by ONE was not found. Interestingly, we found that genes involved in adipocyte browning such as UCP1 and SIRT1 were upregulated by ONE. Since UCP1 and SIRT1 are major proteins expressed in BAT wherein chemical energy is dissipated as heat, adipocyte browning could partly contribute to decreased WAT after treatment with ONE [14]. The degree of upregulation of browning genes was comparable to that in the fenofibrate group. Thus, ONE can inhibit adipocyte hypertrophy in a similar manner and degree to PPARα agonist.

In conclusion, fermented ONE can protect HFD mice against early stage of diet-induced hepatosteatosis and activate FAO via SPHK2 upregulation. ONE can also activate expression of genes in browning genes expression in WAT which plays an important role in reduced adipocyte hypotrophy. Therefore, ONE can be applied for the development of health-promoting beverage or functional food to ameliorate obesity and hepatosteatosis.

## Figures and Tables

**Table 1:** Plasma biochemical parameters of mice with various treatments.

	NCD	HFD	Feno	ON188-50	ON188-100	ON188-200
Plasma glucose (mg/dL)	132.43 ± 4.34 ^a^	136.14 ± 7.71 ^a^	109.14 ± 6.97 ^b^	136.14 ± 6.98 ^a^	143.75 ± 5.55 ^a^	126.50 ± 9.79 ^a^
Cholesterol (mg/dL)	116.26 ± 3.92 ^a^	187.96 ± 10.68 ^b^	138.24 ± 13.24 ^c^	178.54 ± 10.56 ^b^	186.54 ± 6.79 ^b^	173.22 ± 5.99 ^b^
Triglyceride (mg/dL)	80.88 ± 3.74 ^a^	104.92 ± 1.78 ^b^	74.74 ± 3.66 ^a^	105.70 ± 2.96 ^b^	131.44 ± 7.87 ^c^	125.38 ± 6.64 ^c^
HDL (mg/dL)	69.10 ± 3.92 ^a^	106.50 ± 6.92 ^b^	86.56 ± 7.64 ^c^	120.42 ± 6.19 ^b^	118.18 ± 3.99 ^b^	114.24 ± 3.35 ^b^
LDL (mg/dL)	27.50 ± 13.04 ^a^	39.98 ± 15.60 ^a^	36.94 ± 6.34 ^a^	43.28 ± 16.15 ^a^	44.46 ± 21.48 ^a^	41.32 ± 19.40 ^a^
LDH (U/L)	744.36 ± 94.3 ^a^	820.20 ± 102.7 ^a^	704.76 ± 104.2 ^a^	487.56 ± 93.3 ^a^	302.92 ± 48.5 ^b^	163.26 ± 15.2 ^c^
ALT (U/L)	82.96 ± 17.4 ^a^	159.02 ± 25.8 ^b^	105.94 ± 38.7 ^bc^	82.96 ± 29.3 ^c^	83.76 ± 41.7 ^c^	43.46 ± 9.2 ^cd^
AST (U/L)	100.78 ± 18.7 ^a^	180.10 ± 28.8 ^b^	91.20 ± 21.9 ^c^	60.82 ± 15.7 ^c^	71.94 ± 10.4 ^c^	59 ± 8.3 ^c^

Data are means ± standard error to the mean (SEM). One-way analysis of variance (ANOVA) with Bonferroni’s multiple comparison post-hoc analysis. Different letters indicate the statistical significance among groups.

## Supplementary Materials

The following are available online at https://www.mdpi.com/2072-6643/11/5/1015/s1, Table S1: Primer sequences used in this study.
