# Supplementary material for: Fermented Cordyceps militaris Extract Prevents Hepatosteatosis and Adipocyte Hypertrophy in High Fat Diet-Fed Mice
Source: Nutrients. 2019 May 6;11(5):1015. doi: 10.3390/nu11051015 (PMC6566621; doi:10.3390/nu11051015)
Supplement: Supplementary file 1 [file nutrients-11-01015-s001.pdf]

Table S1. Primer sequences used in this study.

Gene	Direction	Sequences (5'→3')
GAPDH	Sense	CAA GGT CAT CCA TGA CAA CTT TG
	Antisense	GGC CAT CCA CAG TCT TCT GG
SPHK2	Sense	AGA CGG GCT GCT TTA CGA G
	Antisense	CCT GCT CAA ACC CGC CAT
CPT1α	Sense	CTT CCA AGG CAG AAG AGT GGG
	Antisense	GAA CCT TGG CTG CGG TAA GAC
PPARα	Sense	ATC CAC GAA GCC TAC C
	Antisense	CAC ACC GTA CTT TAG CAA G
ACOX1	Sense	ACG CCA CTT CCT TGC TCT TC
	Antisense	AGA TTG GTA GAA ATT GCT GCA AA
DGAT2	Sense	CCG CAA AGG CTT TGT GAA
	Antisense	GGA ATA AGT GGG AAC CAG ATC AG
UCP1α	Sense	GTG AAG GTC AGA ATG CAA GC
	Antisense	AGG GCC CCC TTC ATG AGG TC
FGF21	Sense	CTG GGG GTC TAC CAA GCA TA
	Antisense	CAC CCA GGA TTT GAA TGA CC
PRDM16	Sense	CAG CAC GGT GAA GCC ATT C
	Antisense	GCG TGC ATC CGC TTG TG
SIRT1	Sense	CAG TGT CAT GGT TCC TTT GC
	Antisense	CAC CGA GGA ACT ACC TGA T
SREBP-1c	Sense	CGG AAG CTG TCG GGG TAG
	Antisense	GGC CAG AGA AGC AGA AGA GA
aP2	Sense	GAA CCT GGA AGC TTG TCT TCG
	Antisense	ACC AGC TTG TCA CCA TCT CG
PPARγ	Sense	GAG TGT GAC GAC AAG ATT TG
	Antisense	GGT GGG CCA GAA TGG CAT CT
c/EBPα	Sense	GAA CAG CAA CGA GTA CCG GGT A
	Antisense	GCC ATG GCC TTG ACC AAG GAG
